# Effects of increasing temperature and, CO_2_ on quality of litter, shredders, and microorganisms in Amazonian aquatic systems

**DOI:** 10.1371/journal.pone.0188791

**Published:** 2017-11-30

**Authors:** Renato Tavares Martins, Renan de Souza Rezende, José Francisco Gonçalves Júnior, Aline Lopes, Maria Teresa Fernandez Piedade, Heloide de Lima Cavalcante, Neusa Hamada

**Affiliations:** 1 Coordenação de Biodiversidade, Instituto Nacional de Pesquisas da Amazônia—INPA, Manaus, Amazonas, Brazil; 2 Programa de Pós-graduação em Ciências Ambientais, Universidade Comunitária Regional de Chapecó - Unochapecó, Chapecó, Santa Catarina, Brazil; 3 AquaRiparia, Departamento de Ecologia, Instituto de Biologia, Universidade de Brasília, Brasília, Distrito Federal, Brazil; 4 Grupo MAUA ‘‘Ecologia, Monitoramento e Uso Sustentável de Áreas Úmidas”, Instituto Nacional de Pesquisas da Amazônia, Manaus, Amazonas, Brazil; 5 Laboratório de Ecologia, Pós-Graduação em Biologia Urbana, Universidade Nilton Lins, Manaus, Amazonas, Brazil; University of Oklahoma, UNITED STATES

## Abstract

Climate change may affect the chemical composition of riparian leaf litter and, aquatic organisms and, consequently, leaf breakdown. We evaluated the effects of different scenarios combining increased temperature and carbon dioxide (CO_2_) on leaf detritus of *Hevea spruceana* (Benth) Müll. and decomposers (insect shredders and microorganisms). We hypothesized that simulated climate change (warming and elevated CO_2_) would: i) decrease leaf-litter quality, ii) decrease survival and leaf breakdown by shredders, and iii) increase microbial leaf breakdown and fungal biomass. We performed the experiment in four microcosm chambers that simulated air temperature and CO_2_ changes in relation to a real-time control tracking current conditions in Manaus, Amazonas, Brazil. The experiment lasted seven days. During the experiment mean air temperature and CO_2_ concentration ranged from 26.96 ± 0.98ºC and 537.86 ± 18.36 ppmv in the control to 31.75 ± 0.50ºC and 1636.96 ± 17.99 ppmv in the extreme chamber, respectively. However, phosphorus concentration in the leaf litter decreased with warming and elevated CO_2_. Leaf quality (percentage of carbon, nitrogen, phosphorus, cellulose and lignin) was not influenced by soil flooding. Fungal biomass and microbial leaf breakdown were positively influenced by temperature and CO_2_ increase and reached their highest values in the intermediate condition. Both total and shredder leaf breakdown, and shredder survival rate were similar among all climatic conditions. Thus, low leaf-litter quality due to climate change and higher leaf breakdown under intermediate conditions may indicate an increase of riparian metabolism due to temperature and CO_2_ increase, highlighting the risk (e.g., decreased productivity) of global warming for tropical streams.

## Introduction

Global temperatures are increasing due to greenhouse-gas emissions from fossil-fuel combustion and from deforestation, and this is an important threat to aquatic systems [1]. The Intergovernmental Panel on Climate Change (IPCC) estimates under its most-likely scenario without mitigation (RCP8.5) that by the year 2100 average mean temperature is “likely” (66% probability) to increase by 4.8°C over the 1996–2005 average, and that carbon dioxide (CO_2_) levels will reach ~1000 parts per million by volume (ppmv) [1]. Because global mean temperature is mainly affected by temperature over the oceans, the expected increases over continents are higher than these values. In Amazonia, year 2100 temperatures in the June-August period would be 6-8°C over the 1996–2005 average under the same scenario ([1], p. 1343). Global warming can also alter rainy events in some regions [2]. In the Amazon Basin, effects of precipitation changes may vary between regions; for example, an increase in rainfall with subsequent flooding is expected in Western Amazonia, and an increased frequency of droughts is expected in the Central and Lower Amazon areas [2]. Therefore, climate change may also alter flood-pulse dynamics and cause changes in the composition of riparian vegetation [3], with consequent alteration of aquatic communities and detritus input in low-order streams [4–6]. Additionally, in freshwater environments flooding results in an increase of water velocity, depth, and hydrological connectivity and may positively affect aquatic fauna and the leaf-breakdown process [7–8].

As a result of global warming, modifications of leaf-morphological traits, structural and secondary compounds (e.g., tannins) in leaves are expected to increase [9–11], reducing nutrient concentrations and decreasing litter palatability for decomposer communities [12]. Thus, climate and litter changes can also modify metabolic activities of decomposer communities, and, consequently, trophic interactions in streams [13–17]. In low-order streams, the availabilities of carbon and nutrients (e.g., nitrogen and phosphorus) for organisms are driven by leaf-litter breakdown [18]. Therefore, global warming can have important effects on aquatic biogeochemical cycles [4, 11, 19]. These facts increase the importance of studies that predict the effects of climate change on communities and on ecological processes (e.g., leaf-litter breakdown) in aquatic ecosystems [20].

In general, increases in abundance, biomass, and activity of invertebrate shredders and microorganisms (mainly fungi and bacteria) accelerate leaf-litter breakdown [21–22]. Leaf-litter trends determine palatability, where lower leaf hardness (resulting from decreases of fiber, lignin and cellulose concentration) and secondary compounds (e.g., polyphenols and tannins), and higher nutrient concentrations, can increase leaf-breakdown rates [21, 23]. Due to high plant diversity in tropical systems, studies of the interactions of shredders, microorganisms and litter quality are fundamental for understanding the leaf-breakdown process (e.g., [24–26]). Microorganism colonization of leaves, which increases nutritional quality, also increases leaf-litter colonization and consumption by invertebrate shredders. However, little information is available with respect to synergistic effects of increased temperature and CO_2_ on decomposers (shredders and microorganisms), especially in tropical ecosystems [27].

Ecological aspects of shredder invertebrates in tropical ecosystems have been tested in experimental studies, mainly using *Phylloicus* caddisflies (e.g., [25, 28–29]). This genus is present in South and Central American countries [30]. Specifically, *Phylloicus elektoros* the species used in our study, has been recorded in Brazil, Peru and Venezuela [30–31]. Larvae of *Phylloicus* feed on leaf litter and build cases using leaf discs, thus increasing leaf-breakdown rates through leaf fragmentation [32]. However, leaf consumption by *Phylloicus* may be negatively influenced by low litter palatability [33], temperatures beyond tolerance level [14, 27], intra-specific competition and predation [25].

Microorganisms are also influenced by climate change and litter quality [34]. In general, fungi and bacteria have higher activity with increased temperature [35], but litter quality effects (mainly from increased atmospheric CO_2_) are not clear. Some studies show a decrease of microorganism biomass and activity resulting from increased leaf structural compounds [34, 36]. However, no difference has been observed in leaves growing under different CO_2_ conditions, and water temperature has been found to be the major factor affecting the colonization process [37]. In a previous study using the same conditions as the present study (experimental chambers; see methodology), but with leaves not grown in simulated conditions of temperature and CO_2,_ Martins et al. [27] reported that fungal biomass was positively affected by litter quality and negatively affected by increases in temperature and CO_2_.

We evaluated the effects of different scenarios combining increased temperature and CO_2_ on leaf detritus and decomposers. We analyzed leaf consumption of *Hevea spruceana* (Benth) Müll. grown under different air temperatures, CO_2_ concentrations and water availabilities, by shredders (*Phylloicus elektoros*) and microorganisms in experimental chambers simulating climate change. In addition, we also assessed shredder survival and fungal biomass (as ergosterol concentration) under simulated conditions. In general, leaf-litter consumption by shredders and microorganisms increases at high temperatures, but after passing the temperature-tolerance level, consumption decreases due to high metabolic cost. Thus, our hypotheses are that simulated climate change: i) decreases leaf-litter quality, ii) decreases survival and leaf consumption by shredders, and iii) increases microbial decomposition and fungal biomass.

## Methodology

### Ethics statement

Fieldwork was carried out with authorization from the Divisão de Suporte às Estações e Reservas (DSER) of the Instituto Nacional de Pesquisas da Amazônia (INPA). Shredders were collected with authorization and approval of the Brazilian Biodiversity Authorization and Information System (SISBIO; Permit 43934–1). Fieldwork did not involve endangered or protected species.

### Microcosms

We performed the experiment in four microcosm chambers (4.05 m × 2.94 m) that simulated air temperature and CO_2_ changes in relation to a real-time control following current conditions in Manaus, Amazonas, Brazil (S1 Fig). In accord with estimates of air temperature and CO_2_ for the year 2100 [38], the chambers were named: i) Control: real-time current conditions; ii) Light: increases of ~1.5°C and ~220 ppmv in relation to the control condition; iii) Intermediate: increases of ~3.0°C in and ~420 ppmv in relation to the control condition; iv) Extreme: increase of ~4.5°C and ~870 ppmv in relation to the control condition (for temperature and CO_2_ concentration, respectively). Note that these dominations, which have been adopted for all work in these chambers, are overly cautious, since the “Extreme” condition (~4.5°C) is based on the most likely scenario for global mean temperature in the absence of mitigation, and, as noted earlier, the temperature in Manaus would increase by 6–8°C in the hottest part of the year under this scenario [1]. For more information regarding the chambers see [27, 39–40]. Due to the high cost of building and maintaining chambers with real-time variations in air temperature and CO_2_, our design was not completely orthogonal (see more details in [41]). Accordingly, a potential malfunction or any other problem affecting a single chamber would affect all arenas and their growing plants [27]. However, to avoid this potential problem, values of temperature and CO_2_ were recorded every 2 min in all chambers and have high precision [27]. The microcosm was constructed by BIOTEC following instructions for a vivarium cleanroom system with adaptations to increases of temperature and CO2.

### Leaf-litter

In this experiment (see S1 Protocol and S1–S4 Figs), we used leaves of *Hevea spruceana* that were grown under different scenarios combining increased temperature and CO_2_. In the four experimental chambers, plants were subjected to field-capacity soil (non-flooded soil) and flooded soil, the latter with seedlings being flooded up to 5 cm above the soil level. *Hevea spruceana* is a medium-sized rubber tree (up to ~ 27-m height) found on floodplains in the Amazon region [42]. Seeds of *H*. *spruceana* are important food resources for adult fish, among others, including the commercially important “tambaqui”, *Colossoma macropomum*. Moreover, *H*. *spruceana* can be used for rubber production, although it is of relatively poor-quality owing to high proportions of resins [42].

Seedlings (six months) of *H*. *spruceana* were collected in riparian vegetation along the Tarumã-Mirim stream, a tributary of the Rio Negro (03°00’27.47” S, 60°12’14.97 W). Thereafter, plants were acclimatized for a month in a greenhouse and subsequently distributed among the four climatic chambers (Control, Light, Intermediate, and Extreme). Twenty plants were placed in each chamber, which were divided into two soil conditions (flooded and non-flooded). Thus, we had eight initial treatments: 4 chambers × 2 soil conditions. Seedlings were subjected to treatments over a 115-day period, during which fallen leaves were collected. Leaves in all treatments were separately air-dried and stored in an air-conditioned room (20°C). All leaves from each treatment (n = 8) were mixed. We adopted this strategy because plants in each treatment were closely spaced. However, the plants growing on different soil but in the same chamber were spatially isolated. We determined leaf chemical composition using leaves that had not been incubated in the stream (non-leached and non-conditioned). Organic carbon (%) was estimated from the ash-free dry mass. Nitrogen concentration (N; %) was obtained from dry combustion and gas chromatography mass spectrometry analysis [43]. Phosphorus concentration (P; %) was measured by nitric-perchloric digestion and subsequently, determined by spectrophotometry [43]. Cellulose and lignin were measured gravimetrically using acetone and sulfuric acid [44].

Before starting the leaf-breakdown experiment, air-dried leaves of *H*. *spruceana* were incubated for 14 days in the Barro Branco stream (Reserva Ducke—02°55' to 03º01'S; 59º53' to 59º59'W) using litter bags with fine mesh (0.5 mm) in order to leach soluble compounds and allow conditioning by microorganisms [18]. We incubated leaves in the stream to allow natural conditioning before the leaves were broken down by shredders and microorganisms. The stream has dense riparian forest, acidic water (pH = 4.6 ± 0.1), high dissolved oxygen concentration (6.6 ± 0.1 mg l^-1^), low electrical conductivity (10.7 ± 0.4 αS cm^-1^) and mean temperature of 24.5 ± 0.5°C [29]. The leaves were then cut into discs (14 mm in diameter), avoiding the midrib.

### Shredders

We performed the experiment with *P*. *elektoros* larvae. This species is an important shredder in Amazon streams [29]. The larvae are usually abundant in pool mesohabitats [30], favoring their use in laboratory experiments. Larvae of *P*. *elektoros* were also collected from the Barro Branco stream. We collected 120 individuals manually, avoiding last-instar individuals. Subsequently, individuals, were acclimatized for 48 h in plastic containers containing calcined sand (burned at 450°C for 4 h), bottled water (ÁguaCrim®), and partially decomposed leaves from the stream [27].

### Experiment

The experiment lasted seven days, and we used arenas (plastic bottles 11.95-cm height × 9.80-cm diameter; 700-ml volume) with bottled water (500 ml), calcined sand (~ 1 cm height) and constant aeration. We had 15 replicates for each treatment, totaling 120 arenas (4 chambers × 2 soil conditions × 15 replicates). In each chamber, we used only leaves that had grown under the temperature and CO_2_ conditions of the chamber. For example, in the Control chamber, we had 30 arenas with leaf discs from plants grown under control conditions (flooded soil = 15; non-flooded soil = 15; see Appendix). Each arena had disks for only one treatment.

We included five disks in a single treatment in each arena. These disks were used to obtain the total leaf-breakdown rate (by shredders and microorganisms) and were placed on the sands with pins. Six arenas for each treatment had litter bags attached (10 cm × 10 cm; 0.5-mm mesh) with five disks to estimate the leaf-breakdown rate by microorganisms, totaling 12 litter bags per chamber. The disks from the litter bags were used to estimate fungal biomass by ergosterol, filtering with methanol and potassium hydroxide, subsequently analyzed by high-performance liquid chromatography (HPLC), in accord with [45]. Only one *P*. *elektoros* individual was placed in each arena to avoid intraspecific interactions (e.g., cannibalism; [25]). Arenas were inspected daily to verify shredder survival. Arenas where the shredder pupated or died were not considered in the estimates of shredder survival (pupae) or of leaf-breakdown rate.

The leaf-breakdown rate was estimated by mass loss between the initial and final mass [27]. Leaf disks were freeze dried until constant mass (~ 24 hours) and weighed on a precision balance (accuracy = 10 αg). Total leaf-breakdown rate was calculated by dividing the ingested leaf mass (initial dry mass—final dry mass) by the exposure time. Microbial leaf-breakdown rate was obtained by dividing the difference between the initial and final masses of the leaves from the litter bags (not accessible to shredders) by the exposure time. Shredder leaf-breakdown rate was calculated by the difference between total and microbial leaf breakdown. All leaf-breakdown rates were expressed in mg day-1.

We assessed the values of dissolved oxygen (mg l^-1^; oximetry TSI, model 55), pH (WTW, model PH90) and electrical conductivity (μS cm^-1^; WTW, model LF90) in the water in three arenas for each treatment, totaling 24 records per day. In addition, water temperature was recorded each hour using data loggers.

### Statistical analysis

All data were tested for normality and homogeneity using Kolmogorov-Smirnov and Levene’s tests, respectively, and, when necessary, data were log transformed. Paired t-tests were used to test the chemical composition of leaf litter between different soil conditions (flooded and non-flooded). We did not find differences in chemical composition between soils conditions (see Results). Thus, we used data from flooded and non-flooded soils together to test differences of leaf breakdown rates, fungal biomass and P. *elektoros* survival.

We used a linear regression to evaluate the relationship between chemical composition (carbon, nitrogen and phosphorus concentrations) and climate change (Principal Component Analysis–Axis 1 using temperature and CO_2_ data). We used a Repeated Measures Analysis of Variance (RM-ANOVA) to test differences in abiotic variables among simulated climatic conditions (control, light, intermediate, and extreme). One-Way ANOVA was used to test differences in breakdown rates (total, microbial and shredders) and fungal biomass among simulated climatic conditions. We calculated the median time to death (TTD) for each treatment using the Kaplan–Meier product-limit method with the log-rank test [46]. Additionally, survival curves were evaluated using a Chi-square test [46]. All statistical analyses were performed in R software.

## Results

### Abiotic variables

Mean air CO_2_ concentration during the experiment ranged from 537.86 ± 18.36 ppmv in the Control to 1363.78 ± 17.99 ppmv in the Extreme chamber (Table 1). Mean air temperature ranged from 26.96 ± 0.98ºC in the Control to 31.75 ± 0.50ºC in the Extreme chamber. The mean electrical conductivity was similar in all chambers (RM-ANOVA: F_3,47_ = 1.50; p = 0.227). Mean dissolved oxygen (RM-ANOVA: F_3,47_ = 4.92; p = 0.005) and pH (RM-ANOVA: F_3,47_ = 47.36; p < 0.001) were higher in the control chamber and decreased with increased air temperature and CO_2_ concentration. The mean water temperature was lower in the Control chamber (RM-ANOVA: F_3,47_ = 44.10; p < 0.001) and ranged from 25.32 ± 0.41°C in the Control to 29.62 ± 0.37°C in the Extreme treatment. Water abiotic variables were similar between arenas with discs from plants growing in flooded and non-flooded soils (Table 1).

**Table 1:** Mean values (standard error) and results of abiotic variables under simulated climate conditions during seven days of the experiment.

	Abiotic variables	Simulated climate conditions					RM-ANOVA	
		Con	Lig	Int	Ext	df	F	p
**Air**	**CO**_**2**_	537.86±18.36^a^	750.98±16.07^b^	953.13±24.73^c^	1363.78±17.99^d^	**3,18**	**3335.00**	**<0.001**
	**T**	26.96±0.98^a^	28.23±0.96^b^	29.27±0.92^c^	31.75±0.50^d^	**3,18**	**475.90**	**<0.001**
**Water**	**EC**	121.13±14.46^a^	140.69±10.86^a^	149.41±38.30^a^	142.16±57.05^a^	**3,47**	1.50	0.227
	**O**_**2**_	6.70±0.54^a^	6.36±0.64^ab^	6.28±0.32^b^	6.03±0.42^b^	**3,47**	**4.92**	**0.005**
	**pH**	7.47±0.34^a^	7.25±0.28^ab^	6.90±0.16^b^	6.85±0.24^b^	**3,47**	**47.36**	**<0.001**
	**T**	25.32±0.41^a^	26.55±0.35^ab^	27.36±0.56^b^	29.62±0.37^c^	**3,47**	**44.10**	**<0.001**

In bold, p values < 0.05. AV = Abiotic variables; CO_2_ = Carbon dioxide (ppmv); T = Temperature (^o^C); EC = Electrical conductivity (μS cm^-1^); O_2_ = Oxygen (mg l^-1^). Con = Control; Lig = Light; Int = Intermediate; Ext = Extreme.

Different letters (a-d) indicate significant differences between groups.

### Leaf-litter chemical concentration

We did not find significant differences in leaf percentages of carbon, nitrogen, phosphorus, cellulose and lignin (p > 0.223; Table 2) between plants growing on flooded and non-flooded soils. The phosphorus concentration in the leaf litter decreases with increases in temperature and CO_2_ (F_1,6_ = 14.73; R^2^ = 0.66; p = 0.009) and ranged from 0.43 ± 0.08% in the control to 0.20 ± 0.04 in the extreme chamber. Carbon (F_1,6_ = 3.01; R^2^ = 0.22; p = 0.133), nitrogen (F_1,6_ = 0.16; R^2^ = 0.14; p = 0.705), cellulose (F_1,6_ = 0.25; R^2^ = 0.12; p = 0.635), and lignin (F_1,6_ = 3.73; R^2^ = 0.28; p = 0.102) concentrations were not related to the simulated climate change.

**Table 2:** Chemical characteristics of *Hevea spruceana* leaves growing under different conditions of temperature, CO_2_ (Carbon dioxide) and humidity availability. We included paired t-tests for leaf characteristics of plants grown on flooded and non-flooded soils.

	Flooded				Non-flooded				Paired t-test		
	Con	Lig	Int	Ext	Con	Lig	Int	Ext	df	t	p
Carbon (%)	54.56	51.29	51.23	52.34	53.12	52.46	51.76	51.29	3	-0.54	0.626
Nitrogen (%)	1.67	3.85	3.76	2.54	1.99	2.14	2.27	2.26	3	1.18	0.323
Phosphorus (%)	0.37	0.48	0.39	0.17	0.49	0.35	0.28	0.23	3	0.79	0.487
Cellulose (%)	26.95	22.07	24.5	26.78	22.85	26.25	24.04	24.44	3	-0.40	0.716
Lignin (%)	28.13	33.31	28.21	25.83	31.14	27.3	29.89	26.24	3	1.53	0.223

Con = Control; Lig = Light; Int = Intermediate; Ext = Extreme.

### Fungal biomass

**Fig 1:**
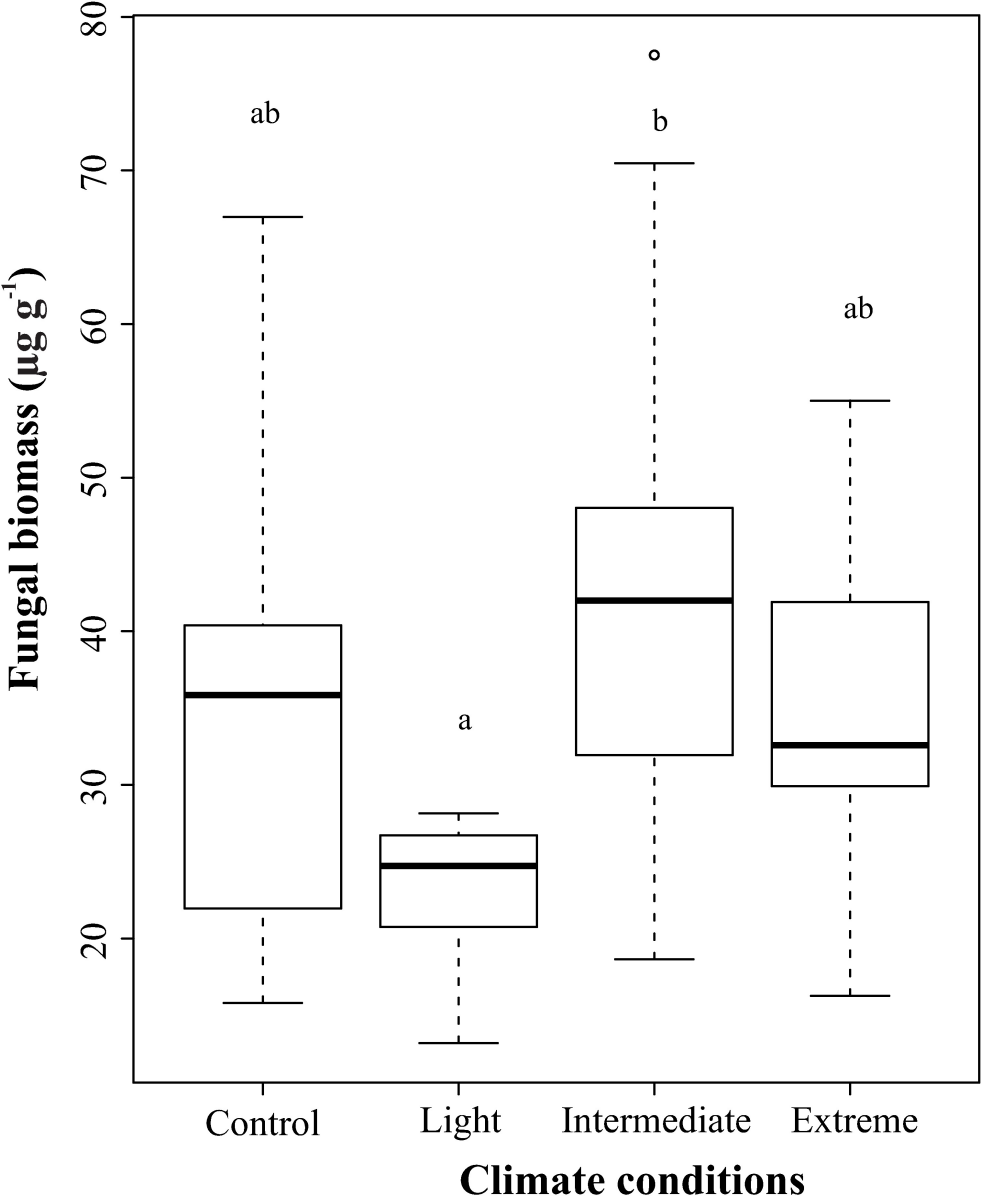
Fungal biomass in treatments with leaf disks of *Hevea spruceana* under four climate conditions during the experiment in Control, Light, Intermediate and Extreme treatments. First (lower line) and third (higher line) quartile, the median (bold line), upper and lower limits (dashed line) and outliers (circles). Highest values = a; lowest values = b (p < 0.05).

The mean value of fungal biomass (ergosterol) ranged from 23.06 ± 4.75 μg g^-1^ in the Light to 42.85 ± 18.03 μg g^-1^ in the Intermediate condition (Fig 1). We found higher fungal biomass in the Intermediate conditions as compared to the other conditions (ANOVA: F_3,45_ = 4.74; p = 0.006).

### Survival

**Fig 2:**
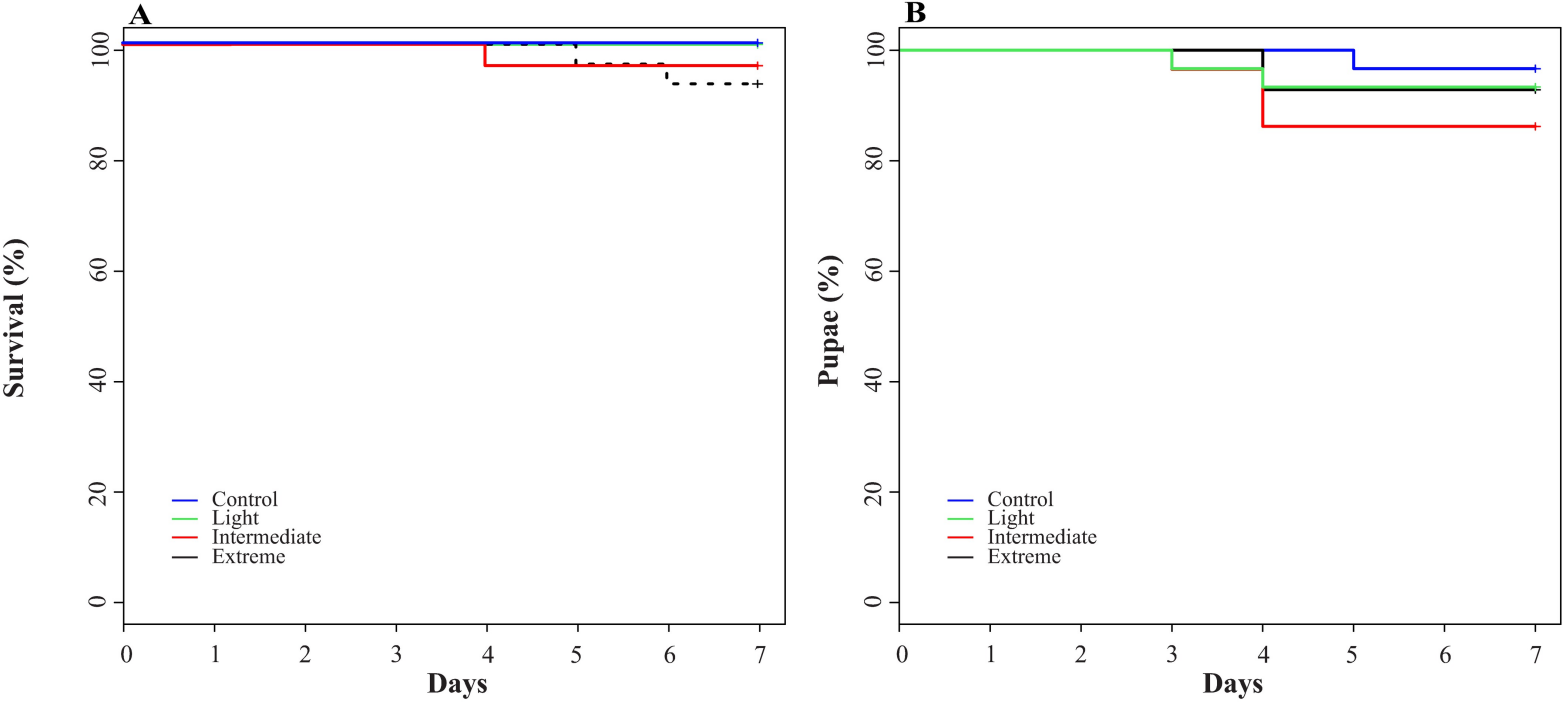
**Survival (%; A) and pupae (%; B) of *Phylloicus elektoros* (Trichoptera: Calamoceratidae) in treatments with leaf disks of *Hevea spruceana* under four climate conditions during the experiment in Control, Light, Intermediate and Extreme treatments**.

We did not find any *P*. *elektoros* death in the control and light chambers. Only three (2.5%) *P*. *elektoros* out of the 120 initial individuals died during the experiment (Fig 2). Survival rates were similar in all treatments (Log-rank, test statistic = 3.7, df = 3, p = 0.290), and were not affected by increased temperature and CO_2_. In addition, pupae were recorded in all treatments (n = 9; Fig 2), and their number was not found to be affected by climate conditions (Log-rank, test statistic = 2.4, df = 3, p = 0.492).

### Leaf-breakdown rate

**Fig 3:**
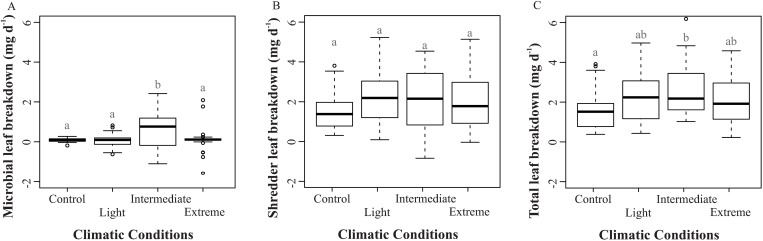
**Shredder (A), microbial (B) and total (C) leaf-breakdown rates in treatments with leaf disks of *Hevea spruceana* under four climate conditions during the experiment in Control, Light, Intermediate and Extreme treatments.** First (lower line) and third (higher line) quartile, the median (bold line), upper and lower limits (dashed line) and outliers (circles). Lowest values = a; Mean values = ab; Highest values = b (p < 0.05).

Under all climate conditions, leaf breakdown was mainly by shredders (Control = 95.15%; Light = 99.11%; Intermediate = 83.46%; Extreme = 93.36%; Fig 3). However, under Intermediate conditions, microorganisms (22.31%) had a key role in leaf breakdown rate. Microbial leaf-breakdown (ANOVA: F_3,113_ = 5.55; p = 0.001) and total leaf-breakdown (ANOVA: F_3,102_ = 2.79; p = 0.044) rates were higher under the Intermediate condition, as compared to the other conditions. On the other hand, leaf breakdown by shredders was similar under all climate conditions (ANOVA: F_3,102_ = 1.48; p = 0.224; Fig 3).

## Discussion

### Leaf-litter chemical concentration

We did not find any difference in the chemical composition of *H*. *spruceana* grown on flooded versus non-flooded soil. The effects of flooding on plants may vary according to plant species, flood tolerance, flood time and climatic conditions [8, 11, 47]. The magnitude of the flooding may also play a role. Under natural conditions, seedlings may remain totally flooded for periods of months, according to their position in the flooding gradient [48]. In general, plants in flooded environments absorb less oxygen and nutrients, resulting in lower concentrations of N and P in the leaves and lower assimilation of CO_2_ [49–51]. However, a non-significant effect of floods on the concentration of N in *Salix sericea* Marshall was reported by Lower et al. [52], probably because their experiment was on the partial flooding of plants. The results may also change if the seedlings remain for a longer period in the experimental chambers.

We found a significant difference in the phosphorus (P) concentration in the leaf litter. P showed a negative correlation with increase in temperature and CO_2_ concentration. This fact is an important sign because P may be a limiting nutrient in addition to nitrogen (N), and may play a key role in global carbon storage [53–54]. Several studies have demonstrated that higher CO_2_ concentrations in the air increase wheat root phosphatase activity (organic phosphate mineralization) and increase the inorganic P supply for plant utilization [54]. This creates a positive feedback, increasing biomass production on P-limited soils [53]. However, this depends in part on plant stoichiometric flexibility [55], and we did not find negative feedback (probably due to the increased use of P with the increased rate of metabolism).

Another aspect is the decrease of P concentration by effects of elevated CO_2_. One would expect the same pattern in biomass-C and N [54], but this was not observed. This may indicate negative effects of CO_2_ increase depending on the species studied, and P may play a more pronounced role than N in regulating riparian vegetation growth [55]. Moreover, interaction with other abiotic factors, such as temperature and flooding can significantly alter the response of plants to CO_2_. Thus, the effect of CO_2_ enrichment may not be manifest if stressors are activated that affect plant growth.

### Fungal biomass

Temperature increase may result in either positive [56–57] or negative [58] effects in leaf-litter breakdown under constant conditions of CO_2_. In our study, fungal biomass showed no linear response to increase in temperature and CO_2_, showing lower values in the Light condition and higher in the Intermediate condition. Soil microorganisms showed increases in most C degradation genes under conditions with high CO_2_ concentration [59–60]. Moreover, in our study higher fungal biomass in the Intermediate condition may be associated with optimal temperature in this chamber, resulting in elevation of metabolic activity, reproductive rates, and fungal biomass [61–62]. However, this stimulus to fungi in warmer water (27.36 ± 0.56°C) may be associated with duration of the experiment (7 days), since some fungal species can tolerate higher temperatures for a brief period [61–63]. For example, a previous study in the same microcosm with a duration of 30 days found different responses of fungal biomass for two leaf species [27]. In leaves of *E*. *glabriflora* there was no variation in fungal biomass among the climatic conditions, whereas in *G*. *glabra* there was a decrease of these microorganisms with increased temperature and CO_2_.

Another important aspect for higher fungal biomass in the Intermediate climate condition is the dominant presence of species adapted to warm water in fungal communities in the tropical region, in contrast to temperate ecosystems (~21 to 27°C) [34, 64]. An example is the dominance of *Anguillospora filiformis*, which is normally associated with warm water [64], confirming the positive effect of temperature [34, 62]. This effect indicates that ecological processes in tropical streams may show less change, for example, in microbial species composition, as compared to temperate streams.

### Survival / pupae

We also observed low mortality of *P*. *elektoros* during the experiment (2.5% of initial individuals). Mortality was not affected by increase in temperature [14] and CO_2_, contrary to the finding of Martins et al. [27]. Survival rates of shredders in laboratory experiments are generally associated with leaf quality, with high mortality on low-quality plant species [58]. However, due to similar chemical composition of the litter, the survival rates between plant species did not change. Increase in temperature and CO_2_ also increase metabolic cost (higher respiratory and excretion rates) and decrease digestive enzyme efficiency [27, 65]. These authors found low survival and higher leaf consumption by *P*. *elektoros*, but this was not observed in our study. Moreover, the short duration of our experiment may have favored the low mortality we observed.

### Leaf breakdown rate

Leaf breakdown was mainly by *P*. *elektoros*, but was similar under all climate conditions. This result shows an important participation of shredders in the leaf-litter breakdown process, despite the low density of shredders in many tropical streams [24, 56, 66]. *Phylloicus* larvae are commonly found on submerged leaves, where they obtain their food and the raw materials for the construction of their cases [21, 32]. Another aspect is the large body size of these larvae and high biomass compared to other shredders in tropical streams [66], which increases the individual importance of *Phylloicus* larvae for leaf-litter breakdown [14, 25]. In the larval phase, *Phylloicus* is exclusively a shredder and helps to convert coarse particulate organic matter into fine particulates and dissolved organic matter [32–33]. The similar leaf-litter breakdown rates under all climate conditions by *Phylloicus* larvae (associated with higher importance) show that the occurrence of these organisms in tropical stream ecosystems may increase the ecological resistance in global-warming scenarios [14–15, 56]. On the other hand, Martins et al. [27] recorded a negative effect of climate change on total and shredder leaf breakdown. Thus, the short duration of our study means that our results regarding the survival and importance of shredders in climate change scenarios should be treated with caution.

Microorganisms are the major decomposers in tropical streams due to the low density of shredders [56, 67]. However, microorganisms show higher sensitivity to extrinsic environmental factors (CO_2_ and temperature) in comparison to shredders [34, 18]. These results were contrary to those observed in the same ecosystem when distinct species are compared (*Goupia glabra* and *Eperua glabriflora*) [27], indicating a: i) a species-specific relationship between decomposer community and leaf-litter quality and ii) the necessity of additional studies to clarify the real effects of climate change on the decomposer community (tri-trophic interaction among leaf-litter, fungi and invertebrates) in litter breakdown. The increase of microbial leaf-breakdown and total leaf-breakdown rates under intermediate conditions can indicate an increase of riparian metabolism in this condition [19, 57]. This highlights the risk (e.g. decreased productivity) of global warming for these ecosystems. Extreme factor values (positive or negative) are within a well-defined range (inside or outside) in the physiological tolerance of organisms [68]. Therefore, advanced stages of global warming could lead to a decrease in decomposing activity, compromising nutrient cycling by microorganisms in aquatic ecosystems.

## Conclusions

Plants in riparian vegetation under different environmental conditions (e.g., soil nutrient and water availability) can produce leaf-litter of differing quality [4]. Different leaf-litter qualities can change their use (food and building-case use) by shredders [14, 24]. However, we did not record significant differences in leaf quality between the leaf litter from plants growing on flooded versus non-flooded soil in the same microcosm (differences were only observed among microcosms). On the other hand, we can also infer that breakdown rates have been changed by global warming [56, 58], independent leaf-litter quality (soil conditions or biome). However, the question remains open as to whether leaf-quality changes are due to the effects of climate change or nutrient concentrations between the leaf-litters sources. Since our case could be due to experimental design or to adaption of this species, further studies are needed.

In conclusion, leaf breakdown was mainly by *Phylloicus* but was similar under all climate conditions. However, the finding that there was low mortality and similar shredder leaf consumption under all climate conditions must be carefully interpreted due to the short duration of our experiment and consequently low lethal effect. We need to investigate chronic effects (over a longer time). We found an increase of microorganism biomass and leaf-breakdown rate under Intermediate conditions, showing higher sensitivity to extrinsic environmental factors. These results also indicate an increase in riparian metabolism under intermediate conditions of temperature and CO_2_, highlighting the risk of global warming for tropical streams.

## Supporting information

S1 Protocol Experimental protocol to study leaf breakdown in four climate conditions. (DOCX)

S1 Fig Architectural plan of microcosm chambers. (TIF)

S2 Fig Representation of arena used to perform experiments. This arena was used to obtain total and shredders leaf breakdown rates.
(TIF)

S3 Fig Sample design of shredder and microbial leaf-breakdown rates in treatments with leaf disks of *Hevea spruceana* under four climate conditions during the experiment in the Control, Light, Intermediate and Extreme treatments. (TIF)

S4 Fig Representation of arena with litter bags. This arena was used to obtain microbial, total and shredders leaf breakdown rates.
(TIF)
