# Supplementary material for: Effects of increasing temperature and, CO2 on quality of litter, shredders, and microorganisms in Amazonian aquatic systems
Source: PLoS One. 2017 Nov 30;12(11):e0188791. doi: 10.1371/journal.pone.0188791 (PMC5708753; doi:10.1371/journal.pone.0188791)
Supplement: S1 Protocol — (DOCX) [file pone.0188791.s001.docx]

**Supplementary material**

**S1 Protocol. Experimental Protocol to study leaf breakdown in four climate conditions**

1- Microcosms

1. The experiment was performed in four similar microcosm chambers (4.05 m × 2.94 m) that simulated air temperature and CO_2_ changes in relation to a real-time control following current conditions from Manaus (Amazonas, Brazil; see S1 Fig).
   1. Antechambers have two doors, and each door opens only if the other is closed. This procedure is adopted to maintain the climatic conditions inside the chamber.
   2. P4 = doors to access antechamber and chamber.
   3. P16 = door to access corridor from the microcosm chambers.

**S1 Fig. Architectural plan of microcosm chambers.**

1. Chambers: i) Control: real-time current conditions; ii) Light: increases of ~1.5°C and ~220 ppmv in relation to the control condition; iii) Intermediate: increases of ~3.0°C in and ~420 ppmv in relation to the control condition; iv) Extreme: increase of ~4.5°C and ~870 ppmv in relation to the control condition (for temperature and CO_2_ concentration, respectively).
2. Temperature and CO_2_ values were recorded every 2 min.
3. Humidity was approximately 70% in all chambers.
4. The photoperiod of the chambers was 12 h light: 12 h dark.

2- Plants in the microcosms

1. Seedlings of ca. six months of *Hevea spruceana* collected in the field (Tarumã-Mirim; 03°00’27.47” S, 60°12’14.97 W) and transplanted to plastic pots (2.5 l). Each pot received a single individual with soil taken at the collection site. Pots were transported to a greenhouse (INPA), remaining in acclimation for one month.
2. Seedlings were distributed in the 4 climatic chambers (Control, Light, Intermediate, and Extreme), 20 seedlings per chamber, divided into two soil conditions (inundated and non-inundated; n=10).
3. Treatments were conducted over a 115-day period, during which fallen leaves were collected, air-dried and stored in an air-conditioned room (20^o^C).
4. Leaf chemical composition
   1. We used leaves that had not been incubated in the stream (non-leached and non-conditioned).
   2. Organic carbon (%)
      1. Difference between mass use the ash-free dry mass.
      2. Leaves were oven-dried for 72 h (60^o^C). Posteriorly, leaves were muffled for 4 h (450^o^C).
      3. $Organic carbon \left( \% \right)=46.5 \times(oven-dried mass-muffled mass)$
      4. Wetzel RG, Likens GE. Limnological analyses. New York, Springer-Verlag; 1991.
   3. Nitrogen concentration (%)
      1. dry combustion and gas chromatography mass spectrometry analysis
      2. Malavolta E, Vitti GC, Oliveira SA. Avaliação do estado nutricional das plantas: princípios e aplicações. Piracicaba: Potafos; 1997.
   4. Phosphorus concentration (%)
      1. nitric-perchloric digestion and subsequently, determined by spectrophotometry
      2. Malavolta E, Vitti GC, Oliveira SA. Avaliação do estado nutricional das plantas: princípios e aplicações. Piracicaba: Potafos; 1997.
   5. Cellulose and lignin (%)
      1. measured gravimetrically using acetone and sulfuric acid
      2. Gessner MO. Proximate lignin and cellulose. In Graça MAS, Barlocher F, Gessner MO, editors. Methods to study litter decomposition. Dordrecht: Springer; 2005a. pp. 115-120.

3- Leaf incubation

1. The air-dried leaves from each treatment were placed in litter bags
   1. 10 leaves in each litter bag
   2. Litter bag: mesh opening = 0.5 mm
   3. Litter bags were labeled and leaves from different treatments were placed in separate litter bags
   4. We incubated leaves to leach soluble compounds and for conditioning by microorganisms
2. The leaves were removed from the stream after 14 days
3. After removal of the litter bags from the stream they were placed individually in plastic bags with stream water (enough to cover the leaves)
   1. The leaves were transported to the laboratory in a cooler containing ice
4. In the laboratory, the leaves were removed from the litter bags and discs were cut from the leaves using a cork borer
   1. Disc diameter: 14 mm
   2. We avoided the midribs of the leaves
5. Leaf discs were frozen (24 hours) and subsequently were freeze dried (~ 24 hours)
6. After freeze drying, the leaf discs were weighed on a precision balance (accuracy = 10 µg) to obtain the initial mass

4- Sample of *Phylloicus elektoros* (Trichoptera: Calamoceratidae)

1. Individuals of *Phylloicus elektoros* were sampled in leaf banks in pools in the Barro Branco stream (Reserva Ducke, Manaus, Brazil)
   1. Individuals were sampled manually
   2. We avoided last-instar individuals
      1. Last-instar individuals have morphological modifications
      2. Individuals about to pupate close the end of the case with a silk-like material
2. Individuals were transported to the laboratory in cooler with water and partially decomposed leaves from the stream
3. In the laboratory, individuals were acclimated for 48 h in a plastic container containing calcined sand (burned at 450°C for 4 h), bottled water (ÁguaCrim®), and partially decomposed leaves from the stream
   1. Room temperature = ~25^o^C
   2. Photoperiod = 12 h dark – 12 h light

5- Experiment preparation

1. Weighed discs (n=5) were pinned and inserted in each arena (plastic bottles = 11.95 cm height × 9.80 cm diameter; 700 ml volume; S2 Fig)

**S2 Fig. Representation of arena used to perform experiments. This arena was used to obtain total and shredders leaf breakdown rates.**

1. Each arena contained bottled water (500 ml; ÁguaCrim®), calcined sand (height = ~ 1 cm) and constant aeration (low water movement) by an aquarium air pump.
   1. Calcined sand = muffle furnace (4 h, 550 ^o^C)
   2. We included only one *Phylloicus elektoros* in each arena.
2. In each chamber, we included 15 arenas with leaf discs of plants growing on each soil type (flooded and non-flooded). Each arena contained discs from plants growing in only one soil type
   1. In total, we used 120 arenas (4 chambers × 2 soil conditions × 15 replicates)
3. In each chamber we used only leaves that had been grown under the temperature and CO_2_ conditions of each chamber. For example, in the control chamber we had 30 arenas with leaf discs from plants grown under control conditions (flooded soil = 15; non-flooded soil = 15, see S3 Fig)

**S3 Fig. Sample design of shredder and microbial leaf-breakdown rates in treatments with leaf disks of *Hevea spruceana* under four climate conditions during the experiment in the Control, Light, Intermediate and Extreme treatments.**

1. Arenas were inspected daily to check shredder survival
   1. In general, dead individuals were not clinging to sand and leaf discs, and may be found floating outside the case
   2. Pupated individuals generally attached their cases to the side wall of the arena
2. Arenas where the shredder pupated or died were not considered in the estimates of shredder survival (pupae) or leaf-breakdown rate
3. We removed the individuals from the experiment when they ate 50% or more of the discs offered (visual determination)
4. After removal of the arenas from the experiment, we removed the leaf discs and they were freeze dried (~ 24 h)
5. Microbial leaf breakdown was calculated using leaf discs in litter bags (no shredders access; see S4 Fig)
   1. $Microbial leaf-breakdown=\frac{initial mass-final mass}{exposure time}$
   2. Leaf discs used to calculate microbial leaf breakdown, were also subjected to ergosterol determination (in accord with Gessner, 2005)

**S4 Fig. Representation of arena with litter bags. This arena was used to obtain microbial, total and shredders leaf breakdown rates.**

1. Total leaf breakdown and shredder leaf breakdown were calculated using leaf discs exposed to shredders
   1. $Total leaf-breakdown=\frac{initial mass-final mass}{exposure time}$
   2. $Shredders leaf-breakdown=(total leaf-breakdown)-(Microbial leaf-breakdown)$
2. We made daily measurements of dissolved oxygen (mg l^-1^; oximetry TSI, model 55), pH (WTW, model PH90) and electrical conductivity (µS cm^-1^; WTW, model LF90) of the water in three arenas for each treatment, totaling 24 records per day.
