# Supplementary figures and images for: Effects of increasing temperature and, CO2 on quality of litter, shredders, and microorganisms in Amazonian aquatic systems
Source: PLoS One. 2017 Nov 30;12(11):e0188791. doi: 10.1371/journal.pone.0188791 (PMC5708753; doi:10.1371/journal.pone.0188791)

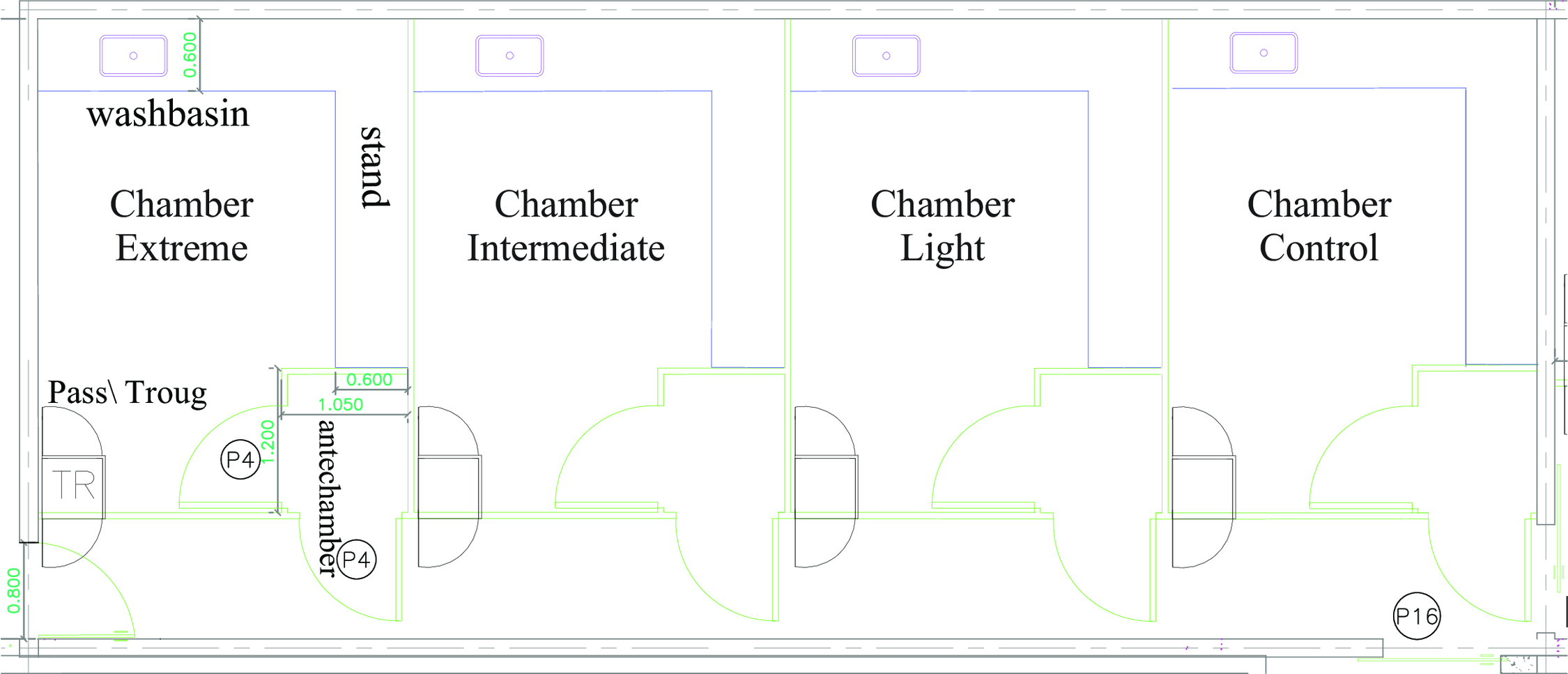

Supplement: S1 Fig — (TIF) [file pone.0188791.s002.tif]

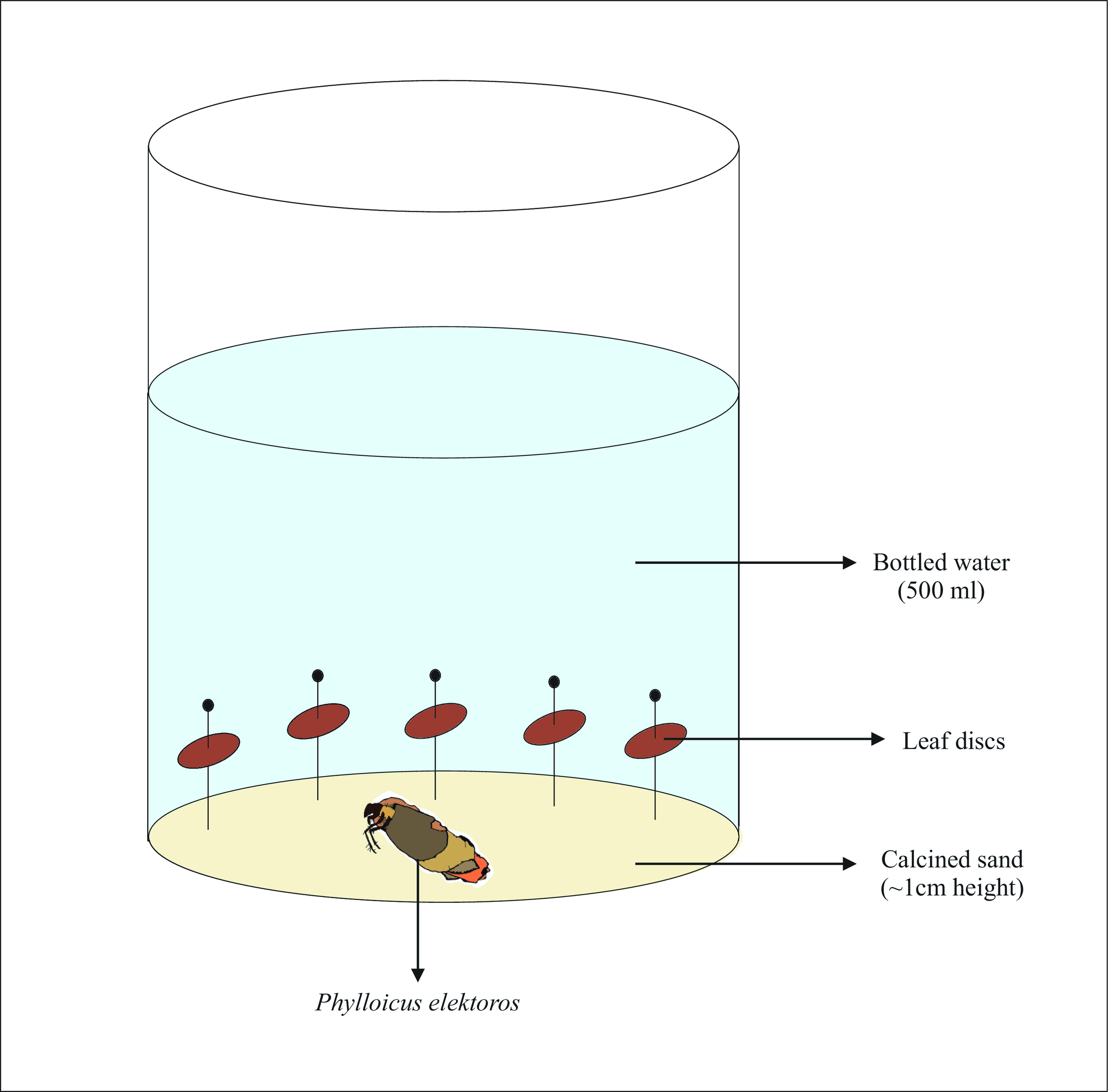

Supplement: S2 Fig — This arena was used to obtain total and shredders leaf breakdown rates. (TIF) [file pone.0188791.s003.tif]

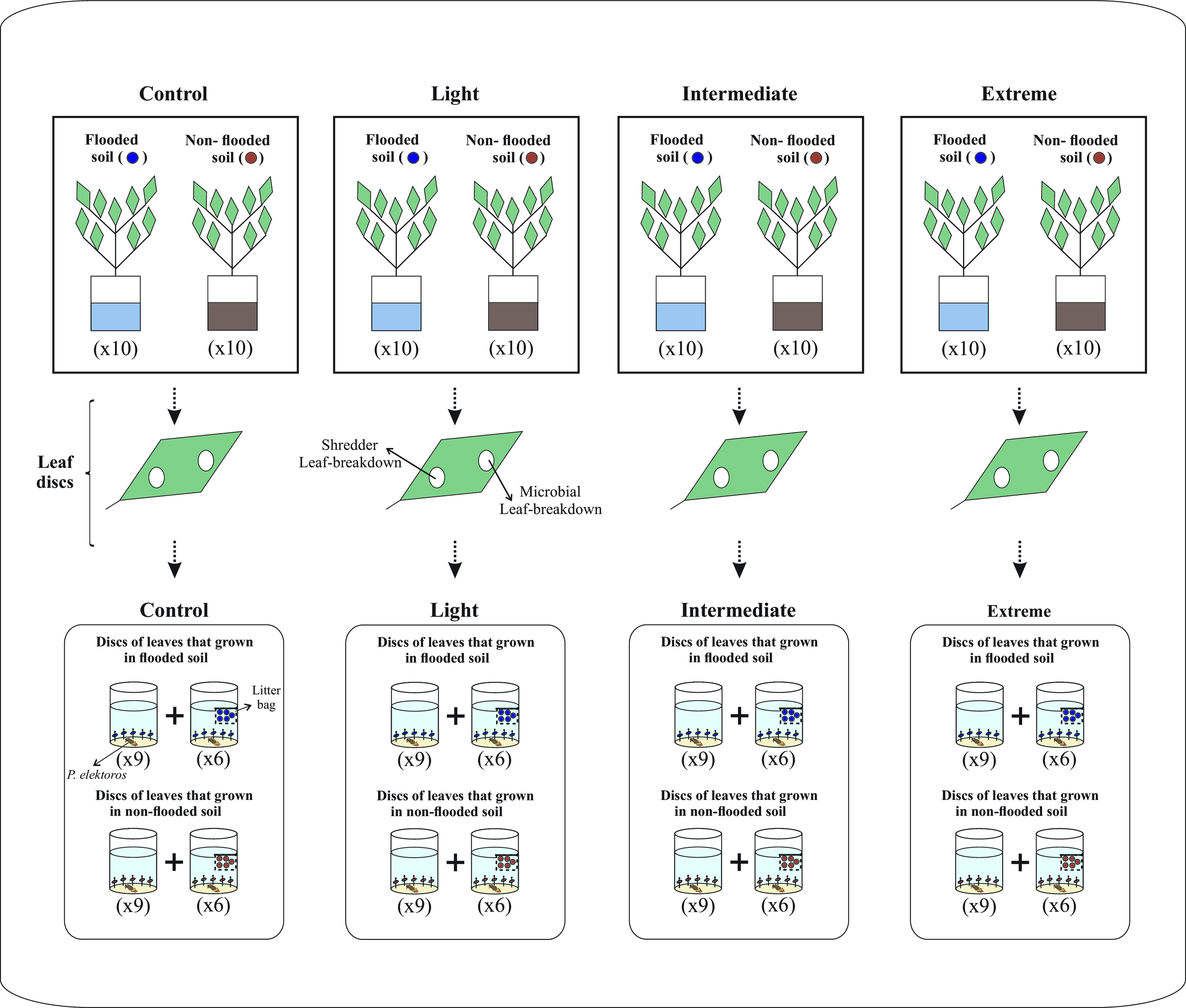

Supplement: S3 Fig — (TIF) [file pone.0188791.s004.tif]

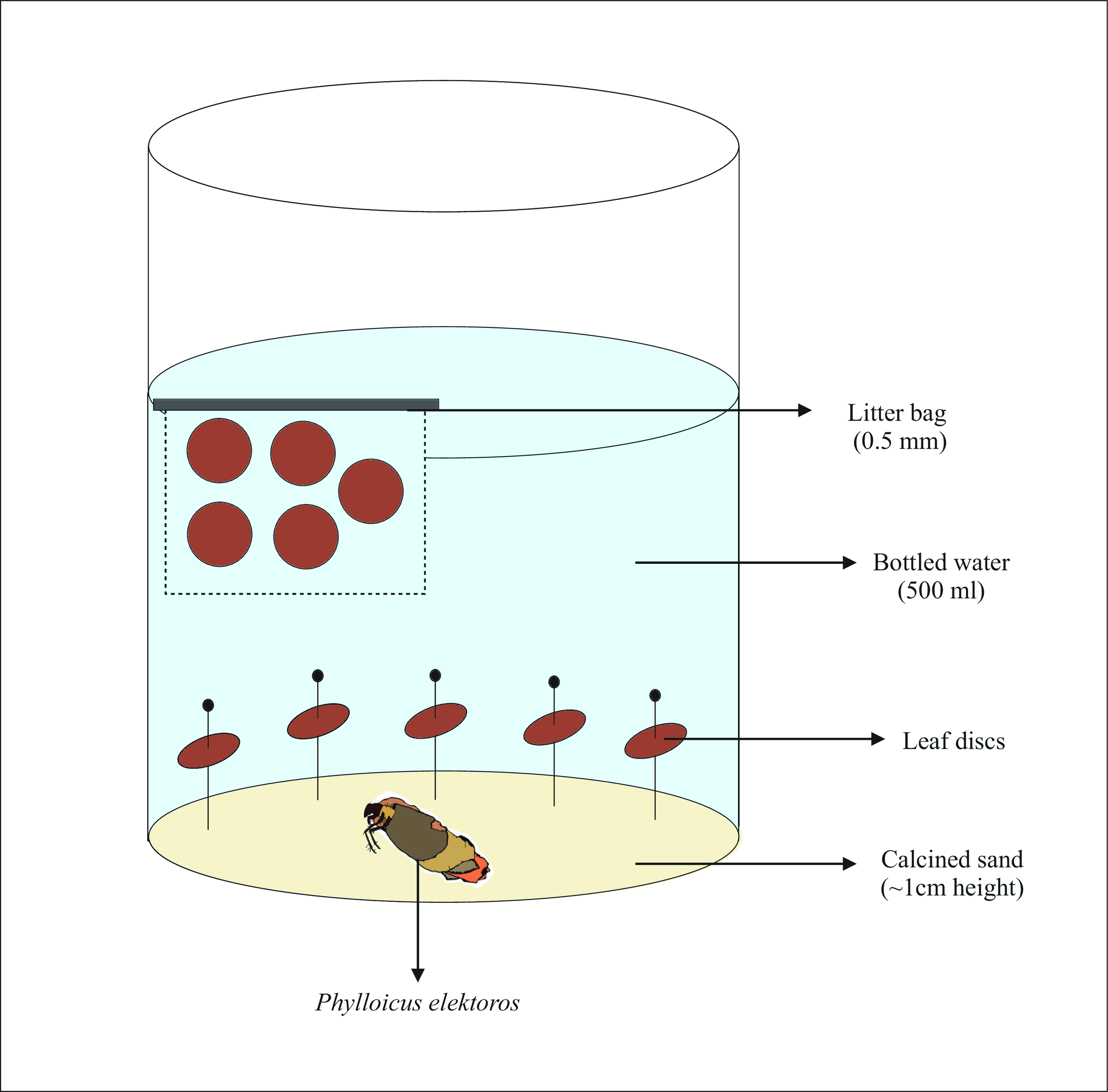

Supplement: S4 Fig — This arena was used to obtain microbial, total and shredders leaf breakdown rates. (TIF) [file pone.0188791.s005.tif]
